# Virtual Reality-Based Therapy Improves Fatigue, Impact, and Quality of Life in Patients with Multiple Sclerosis. A Systematic Review with a Meta-Analysis

**DOI:** 10.3390/s21217389

**Published:** 2021-11-06

**Authors:** Irene Cortés-Pérez, Marcelina Sánchez-Alcalá, Francisco Antonio Nieto-Escámez, Yolanda Castellote-Caballero, Esteban Obrero-Gaitán, María Catalina Osuna-Pérez

**Affiliations:** 1 Granada Northeast Health District, Andalusian Health Service, Street San Miguel 2, 18500 Guadix, Granada, Spain; icp00011@red.ujaen.es; 2 Department of Health Sciences, University of Jaén, Campus Las Lagunillas s/n, 23071 Jaén, Jaén, Spain; msalcala@ujaen.es (M.S.-A.); mycastel@ujaen.es (Y.C.-C.); mcosuna@ujaen.es (M.C.O.-P.); 3 Jaén-Jaén Sur Health District, Andalusian Health Service, Street Cataluña, 23006 Jaén, Jaén, Spain; 4 Department of Psychology, University of Almería, Road Sacramento s/n, 04120 La Cañada, Almería, Spain; pnieto@ual.es; 5 Center for Neuropsychological Assessment and Neurorehabilitation (CERNEP), University of Almería, Road Sacramento s/n, 04120 La Cañada, Almería, Spain

**Keywords:** multiple sclerosis, virtual reality, videogames, fatigue, quality of life, meta-analysis

## Abstract

Patients with multiple sclerosis (PwMS) have a high level of fatigue and a reduced quality of life (QoL) due to the impact of multiple sclerosis (MS). Virtual reality-based therapy (VRBT) is being used to reduce disability in PwMS. The aim of this study was to assess the effect of VRBT on fatigue, the impact of MS, and QoL in PwMS. Methods: A systematic review with meta-analysis was conducted through a bibliographic search on PubMed, Scopus, Web of Science, and PEDro up to April 2021. We included randomized controlled trials (RCTs) with PwMS that received VRBT in comparison to conventional therapy (CT) including physiotherapy, balance and strength exercises, and stretching or physical activity, among others; or in comparison to simple observation; in order to assess fatigue, MS-impact, and QoL. The effect size was calculated using Cohen’s standardized mean difference with a 95% confidence interval (95% CI). Results: Twelve RCTs that provided data from 606 PwMS (42.83 ± 6.86 years old and 70% women) were included. The methodological quality mean, according to the PEDro Scale, was 5.83 ± 0.83 points. Our global findings showed that VRBT is effective at reducing fatigue (SMD −0.33; 95% CI −0.61, −0.06), lowering the impact of MS (SMD −0.3; 95% CI −0.55, −0.04), and increasing overall QoL (0.5; 95% CI 0.23, 0.76). Subgroup analysis showed the following: (1) VRBT is better than CT at reducing fatigue (SMD −0.4; 95% CI −0.7, −0.11), as well as in improving the mental dimension of QoL (SMD 0.51; 95% CI 0.02, 1); (2) VRBT is better than simple observation at reducing the impact of MS (SMD −0.61; 95% CI −0.97, −0.23) and increasing overall QoL (SMD 0.79; 95% CI 0.3, 1.28); and (3) when combined with CT, VRBT is more effective than CT in improving the global (SMD 0.6, 95% CI 0.13, 1.07), physical (SMD 0.87; 95% CI 0.3, 1.43), and mental dimensions (SMD 0.6; 95% CI 0.08, 1.11) of QoL. Conclusion: VRBT is effective at reducing fatigue and MS impact and improving QoL in PwMS.

## 1. Introduction

Multiple sclerosis (MS) is a chronic, inflammatory, immune-mediated, and currently incurable disease that affects the central nervous system (CNS) [1]. It results in demyelination, glial reaction, and axonal loss [2]. MS is the leading cause of disability by chronic neurological disease in young adults [3], affecting more than 2.5 million people worldwide [4], with a prevalence of 36 cases per 100,000 people [5]. In Europe, MS shows a prevalence of 83 cases per 100,000 habitants, with an average annual incidence of 4.3 cases per 100,000 members of the population [6]. MS more frequently affects females, at a 3:1 ratio before puberty [7] (1:1 after menopause [8]), which has been explained as the result of a higher female predisposition to immune diseases due to chromosomal sex and hormonal susceptibility [9,10]. In recent years, increases in life expectancy have been linked to a rise in the prevalence of MS, resulting in a sizeable socio-economic burden on healthcare systems and communities, with a mean annual cost per patient between 463 USD and 58,616 USD [5].

Patients with MS (PwMS) show a large variety of disabling symptoms that considerably reduce their health-related quality of life (QoL) in comparison to other neurological diseases [11]. MS evolves with different motor, somatosensory, and psychocognitive impairments that reduce a patient’s functional capacity, such as muscle tone disorders [12] including spasticity, a decrease in range of motion and the body’s mobility [13], and an increase in the level of physical fatigue. Fatigue is experienced by 75% of PwMS (ranging between 52 and 88%), and is considered the most disabling MS symptom in PwMS [14]. In addition, muscle fatigue may reduce gait and balance ability in PwMS [15]. Fatigue is also associated with impaired cognitive functioning, reducing work productivity [16], producing psychological disorders such as anxiety or depression, and reducing personal autonomy and social abilities [17].

In addition to pharmacological treatments and non-pharmacological interventions such as conventional therapy (CT), exercise, or complementary therapies traditionally used in PwMS, in the last decade, the advance in digital technologies has boosted the use of new tools such as smartphones, websites, wearables, and virtual reality (VR) devices in neurorehabilitation protocols. VR is a novel technology whose therapeutic and rehabilitative effect is being tested for different CNS diseases [18,19], as well as being used in the practical training of healthcare students [20]. Weiss has defined VR as the “use of interactive simulations created with computer hardware and software to present users with opportunities to engage in environments that appear and feel similar to real world objects and events” [21]. VR-based therapy relies on two concepts: (1) presence (the psychological feeling of being inside a virtual scenario similar to the real world); and (2) immersion (linked to the level of sensory realism and interaction possibilities in the virtual environment) [22]. According to the level of immersion, different modalities of VR may be used in neurorehabilitation, with non-immersive VR (niVR) being the most used VR system in neurorehabilitation to date. In such a case, a virtual scenario is projected onto a screen and patients interact with it by using a mouse or joystick [23]. Nintendo^®^ Wii, Kinect^®^, and games designed for computers or sensors such as Leap Motion^®^ are considered niVR. In addition, VR can be considered semi-immersive when large screens are used [24]. In comparison, immersive VR (iVR) allows a 360° view of a virtual environment through a head-mounted display, in which patients can interact with virtual objects by using hand-held controllers or their own hands [25]. Some examples of this technology are the Oculus Quest or HTC Vive. It has been argued that VR-based therapy promotes neuronal plasticity, modulates synaptic transmission and neuronal excitability, reorganizes synaptic connections and neuronal morphology, and reshapes dendritic spines [26].

VR offers an active, multi-sensory, and fun therapy with immediate feedback that may increase the motivation and adherence of patients to the therapy [27]. PwMS have reported high levels of usability and acceptability regarding the use of VR systems in neurorehabilitation [28]. In relation to the use of VR on combating symptoms of MS, some reviews have assessed its effect on gait and balance [29,30], as well as on motor impairments of the upper extremities [31], with interesting results. Therefore, the aim of this review was to collect all available published evidence that permits us to analyze the effect of VR therapy on fatigue, MS impact, and QoL in PwMS.

## 2. Materials and Methods

### 2.1. Design

The Preferred Reporting Items for Systematic Reviews and Meta-Analyses (PRISMA) guidelines [32] and the Cochrane Handbook for Systematic Reviews of Interventions [33] were used to perform this systematic review with a meta-analysis.

### 2.2. Search Strategy and Data Sources

Two authors (I.C.-P. and E.O.-G.) performed a bibliographic search on PubMed Medline, Scopus, Web of Science (WOS), PEDro (Physiotherapy Evidence Database), and other sources such as previously published reviews, books, practice guidelines, and gray literature (conference proceedings), with search parameters up until April 2021. The keywords used in this search strategy, according to the Medical Subjects Headings (MeSH), were “multiple sclerosis”, “virtual reality”, “virtual reality exposure therapy”, “fatigue”, and “quality of life”. We used the population, intervention, comparison, outcomes, and study (PICOS) tool proposed by Cochrane Collaboration [33]: population (PwMS), intervention (VR), comparison (conventional therapy (CT) or no intervention (NI)), outcomes (fatigue, impact, and QoL) and study design (randomized controlled trial (RCT)). Boolean operators “AND”/“OR” were used. No publication date and language restrictions were applied. A third expertise author (M.C.O.-P.) supervised this stage. Table 1 shows the search strategy employed.

### 2.3. Inclusion Criteria

The study selection stage was carried out by two authors (I.C.-P. and E.O.-G.) who independently screened the titles and abstracts of all studies retrieved by the search strategy from each database. Studies selected by at least one author were considered eligible for inclusion in this systematic review and were reviewed in detail. A third author (F.A.N.-E.) was consulted when a study raised doubts about its inclusion. The inclusion criteria applied were: (1) RCT or RCT pilot; (2) participants were PwMS; (3) the study design included at least two groups; (4) one group received an intervention with VR and the second group CT or NI; (5) the study aimed to assess the effect of VR on fatigue, MS-impact, or QoL; and (6) the study provided quantitative data about the variables of interest for the meta-analysis. The exclusion criterion was RCTs including different neurological diseases apart from PwMS.

### 2.4. Data Extraction

Two authors (I.C.-P. and M.C.O.-P.) independently compiled data from the included studies in a standardized Excel data collection form. Disagreements were resolved by a third researcher (F.A.N.-E.). The following data were extracted: (1) authorship, publication date, study design, country, and funding received; (2) data related to participants (number of PwMS, age and sex); (3) experimental intervention characteristics (VR therapy length in weeks, number of sessions per week, and session time in minutes); (4) type of control intervention; (5) quantitative data obtained at the post-therapy evaluation (mean and standard deviation); and (6) follow-up time (immediate or long-term). Regarding quantitative data, when a study did not provide standard deviation, we estimated this measure using standard error, interquartile range, or range, using standardized transformations according to the Cochrane Handbook for Systematic Reviews of Interventions [33] and previous studies [18].

### 2.5. Outcomes

The outcomes assessed in the present review were: level of fatigue, impact of MS, and QoL. The selected studies provided data from different tests for each outcome (see outcomes section in Results).

### 2.6. Risk of Bias Assessment and Quality Evidence

The PEDro scale was independently used by two authors (M.S.-A. and Y.C.-C.) to assess the risk of bias and the methodological quality of the included studies. The PEDro scale comprises 11 items (item 1 is not used for the total score), with a score ranging from 0 (very low methodological quality and high risk of bias) to 10 (high methodological quality and low risk of bias) [34]. A study was considered high quality if it scored equal to or higher than 8 points [35].

In addition, the level of evidence of each meta-analysis was analyzed using the Grading of Recommendations Assessment, Development, and Evaluation (GRADE) metric. According to Meader (2014) [36], a level of evidence is conditioned by its risk of bias, inconsistency, imprecision, indirectness, and risk of publication bias. Inconsistency was assessed by estimating the level of heterogeneity (see statistical analysis section); imprecision was calculated from the number of participants per study and the number of studies in each meta-analysis, and indirectness was noted in articles in which the results were measured indirectly, registered as a “yes” or “no” [33]. Finally, the level of evidence was scored as follows: (1) high, if findings were robust; (2) moderate, if results might change after including new studies; (3) low, if the level of confidence in our pooled effect was very slight; and (4) very low, when any effect estimation was robust because some of Meader’s items were not present in the studies included in the meta-analysis. Two authors (I.C.-P. and F.A.N.-E.) independently assessed the level of evidence of each meta-analysis and doubts were discussed with a third author (M.C.O.-P.).

### 2.7. Statistical Analysis

A meta-analysis was performed by two authors using Comprehensive Meta-Analysis version 3.0 (Biostat, Englewood, NJ, USA) [37] (E.O.-G. and I.C.-P.). The effect was estimated in a random effect of DerSimonian and Laird [38] using Cohen’s standardized mean difference (SMD) [39] with a 95% confidence interval (95% CI), according the guidelines established by Cooper et al. [40]. Cohen’s SMD can be interpreted as a four-level strength effect: no effect (SMD 0), small (SMD 0.2–0.4), medium (SMD 0.4–0.7) and large (SMD > 0.8) [41]. The result of each meta-analysis was displayed in forest plots [42]. Red diamonds represent the overall results of the meta-analysis, either from the subgroup analysis performed (subtotals) or from the set of all groups (total). The center of the diamond is the overall effect value and the width represents the overall confidence interval. The difference between the intervention and control groups can be considered statistically significant if the diamond is clearly positioned to one side of the reference line, but if it crosses it or just rubs it, no conclusions can be drawn from that point in one direction. The *p*-value for Egger’s test (with *p* < 0.1 showing a risk of publication bias) [43], the visualization of the funnel plot [44] (which in cases of asymmetry indicates a possible risk of publication bias), and trim-and-fill estimation [45] were used to estimate the risk of publication bias. When the trim-and-fill estimation reported a variation higher than 10% with respect to the original pooled effect, the level of evidence was downgraded one level [46]. The level of heterogeneity was calculated by using the Q-test and its *p*-value (*p* < 0.1 indicates the existence of heterogeneity) and the degree of inconsistency (I^2^) established by Higgins [47], where the level of heterogeneity can be rated as low (I^2^ < 25%), moderate (I^2^ between 25–50%), or large (I^2^ > 50%) [47].

### 2.8. Sensitivity Analysis

The leave-one-out method (or one-study-removed method) was employed to assess the contribution or weight of each study to the global effect in each meta-analysis [33].

### 2.9. Subgroup Analysis

A subgroup analysis [33] was conducted to assess the effect of VR according to the comparisons conducted in the included studies. These comparisons showed the following: (1) VR vs. NI; (2) VR vs. CT; and (3) VR + CT vs. CT.

## 3. Results

### 3.1. Study Selection

**Figure 1:**
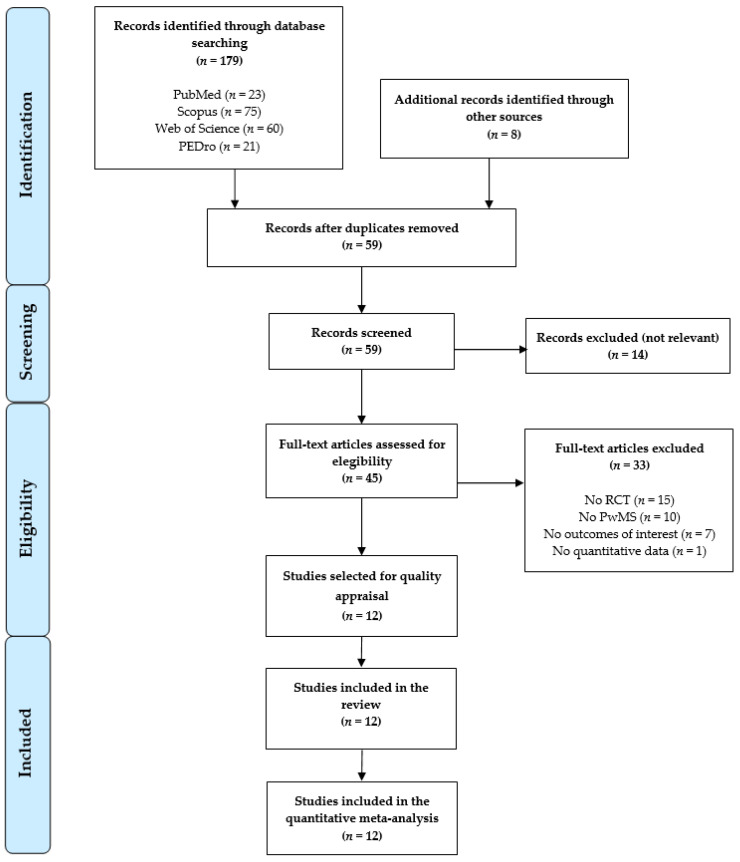
PRISMA (Preferred Reporting Items for Systematic Reviews and Meta-Analyses) flow chart for study selection.

We identified 179 studies from different databases (PubMed *n* = 23, Scopus *n* = 75, WOS *n* = 60, and PEDro *n* = 21) and another eight additional records were identified from other sources. After duplicated studies were removed (*n* = 128), 59 studies were screened by title/abstract. Fourteen studies were excluded in the first screening and thirty-three were excluded afterwards, as they did not meet the inclusion criteria (reasons in Figure 1). Finally, 12 studies were included in the present systematic review with a meta-analysis [48,49,50,51,52,53,54,55,56,57,58,59]. Figure 1 shows the PRISMA flow chart for the study selection process.

### 3.2. Characteristics of the Studies Included

The twelve RCTs included provided 26 independent comparisons (ten for fatigue analysis, eight for impact of MS analysis, and eight for QoL analysis). Supplementary Table S1 summarizes and details the independent comparisons identified in each study. All included studies were RCTs [48,49,50,51,52,53,54,55,56,57,58,59] carried out in the last decade between 2013 and 2020 in Italy [48,55,59], Spain [54], France [50], the UK [56,57], Hungary [58], Turkey [51,52,53], and Jordan [49]. These studies report data from 606 PwMS with a mean age of 42.83 ± 6.86 years. In total, 442 PwMS were female (73%) and 164 were male (27%), and 313 PwMS (70% women with a mean age of 43.45 ± 7.64 years) received an intervention based on VR. Of the 12 studies included, only nine in the experimental group used VR intervention only [48,49,50,51,52,53,55,56,58], while the remaining three used VR and CT [54,57,59]. The VR interventions used in the studies included were as follows: (1) niVR, such as the Nintendo^®^ Wii Balance Board^®^ [48,49,55,57], Nintendo^®^ Wii Fit^®^ [53,56], Leap Motion® [54], REACTIV program [50], Xbox 360^®^ with Kinect® Sensor [58], Xbox One^®^ and Kinect Sensor [51]; (2) semi-immersive VR using the BTS-Nirvana® system [59]; (3) iVR using the RAGU system with Oculus® [52]. By contrast, 293 PwMS (75% women with a mean age of 42.22 ± 6.15 years) were included in a comparison control group receiving CT or simple observation (NI). Regarding intervention length, VR therapies lasted from 4 weeks to 4 months, with a frequency of one to five sessions per week and a time per session ranging from 20 to 60 min. All data from the selected studies were obtained just after the end of the therapy (no long-term follow-up assessment was conducted). Finally, four studies [48,52,55,58] reported that no funding was received to carry out the research, seven studies [49,50,51,53,54,56,57] received funding, and one study did not report such information [59]. Table 2 summarizes the main characteristics of the studies included in this review.

### 3.3. Methodological Quality Assessment

According to the PEDro scale, the mean methodological quality of the included studies was low (mean PEDro score = 5.83 ± 0.83 points). Five studies [48,51,52,53,56] showed low quality and a high risk of bias and seven studies [49,50,54,55,57,58,59] showed medium quality and a moderate risk of bias. Table 3 shows PEDro scores for all included studies.

### 3.4. Outcomes Synthesis

The level of fatigue was analyzed using the Fatigue Severity Scale (FSS) [52,53,54] and the Modified Fatigue Impact Scale (MFIS) [48,49,50,51]; MS impact was assessed with the Multiple Sclerosis Impact Scale-29 (MSIS-29) [54,55,57,58] and the World Health Organization Disability Assessment Schedule 2.0 (WHODAS 2.0) [56]; and QoL was assessed using the Multiple Sclerosis Quality of Life test (MSQoL) [51,53,59] and the SF-36 scale [49,50,57].

### 3.5. Meta-Analysis Findings

#### 3.5.1. Effect of Virtual Reality on Fatigue

**Figure 2:**
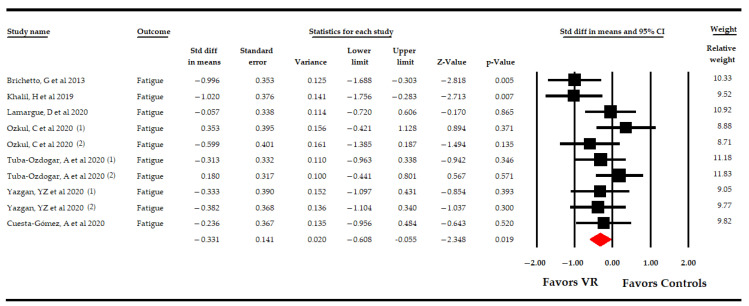
Forest Plot of the Effect of Virtual Reality on Fatigue.

Seven RCTs [48,49,50,51,52,53,54] provided data from 319 PwMS to assess the effect of VR-based intervention on fatigue. At first, an overall analysis showed moderate-quality evidence of a low to medium effect favoring VR-based therapy (SMD −0.33; 95% CI −0.61 −0.06; *p* 0.02) (Table 4, Figure 2) compared to CT or NI, without heterogeneity (I^2^ 0%; Q-test = 8.9, df = 9; *p* 0.44) and no risk of publication bias (*p* for Egger = 0.47) (Supplementary Figure S1). Sensitivity analysis showed a change of 20% after removing a study by Brichetto [51].

**Figure 3:**
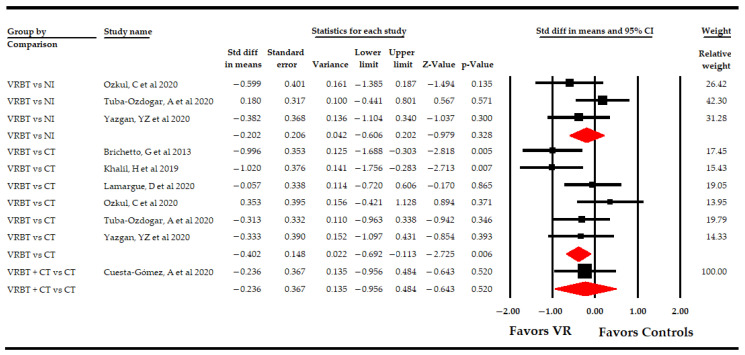
Subgroup Analysis of the Effect of Virtual Reality on Fatigue According to Specific Comparisons.

The included studies showed the following comparisons: VR vs. NI, VR vs. CT, and VR + CT vs. CT (Table 4, Figure 3). Three studies [51,52,53] compared VR-based intervention vs. simple observation, without finding significant differences between groups (SMD −0.2; 95% CI −0.61, 0.2; *p* 0.33), without heterogeneity (I^2^ 0%; Q-test = 1.96, df = 2; *p* 0.37), and with no risk of publication bias (*p* for Egger test 0.1). Six studies [48,49,50,51,52,53] compared VR-based intervention vs. CT, showing moderate-quality evidence of a medium effect favoring VR-based intervention (SMD −0.4; 95% CI −0.7 −0.11; *p* 0.006), without heterogeneity (I^2^ 1.9%; Q-test = 5.1, df = 5; *p* 0.41) and with a possible risk of publication bias (*p* for Egger test 0.46 and 25% of variation after trim-and-fill estimation). Finally, one study [54] compared VR-based intervention with CT vs. CT, without showing significant differences between the two groups (SMD −0.23; 95% CI −0.95, 0.48; *p* 0.52).

#### 3.5.2. Effect of Virtual Reality-Based Therapy on the Impact of Multiple Sclerosis

**Figure 4:**
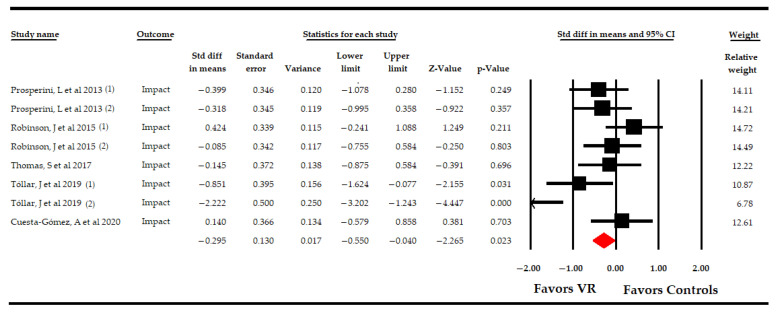
Forest Plot of the Effect of Virtual Reality on the Impact of Multiple Sclerosis.

Five RCTs [54,55,56,57,58] provided data from 287 PwMS to assess the effect of VR-based therapy on the impact of MS in comparison to CT or NI. At first, an overall analysis showed low-quality of a low-to-medium effect favoring VR-based therapy (SMD −0.3; 95% CI −0.55, −0.04; *p* 0.02) on the impact of MS compared to CT or NI (Table 4, Figure 4), with a low level of heterogeneity (I^2^ 21%; Q-test = 8.9, df = 7; *p* 0.26) and a low risk of publication bias (*p* for Egger = 0.07 and 50% of variation after trim-and-fill estimation) (Supplementary Figure S2). Sensitivity analysis reported a variation of 41% with respect to the original effect, excluding a study by Tollár [58].

**Figure 5:**
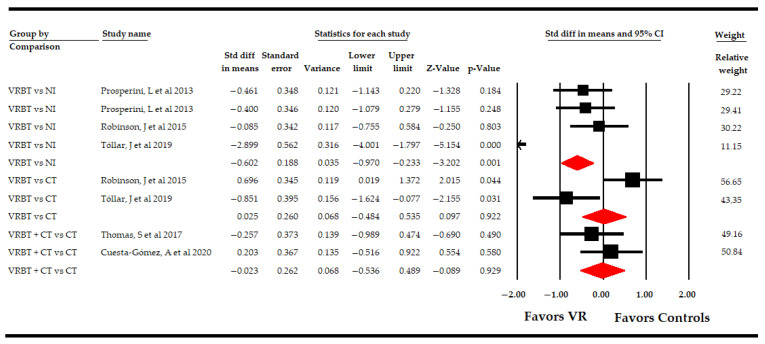
Subgroup Analysis of the Effect of Virtual Reality on the Impact of Multiple Sclerosis According to Specific Comparisons.

The following specific comparison subgroups were identified: VR vs. NI, VR vs. CT, and VR + CT vs. CT (Table 4, Figure 5). Firstly, three studies [56,58] compared the effect of VR-based intervention vs. simple observation, showing low-quality evidence of a medium effect favoring VR-based intervention (SMD −0.61; 95% CI −0.97, −0.23; *p* 0.001), with a low level of heterogeneity (I^2^ 27.7%; Q-test = 5.5, df = 4; *p* 0.23) and taking into account a possible risk of publication bias (*p* for Egger test 0.001 and 23% of variation after trim-and-fill estimation). Secondly, two studies [56,58] reported data assessing the effect of VR-based intervention vs. CT, and no statistically significant differences between groups were observed (SMD −0.03 95% CI −0.48, 0.53; *p* 0.92), without heterogeneity (I^2^ 0%; Q-test = 0.98, df = 1; *p* 0.32). Finally, two studies [54,57] compared VR + CT vs. CT without reporting significant differences between these groups (SMD −0.02; 95% CI −0.54, 0.49; *p* 0.93), without heterogeneity (I^2^ 0%; Q-test = 0.3, df = 1; *p* 0.6).

#### 3.5.3. Effect of Virtual Reality-Based Therapy on Overall Quality of Life

**Figure 6:**
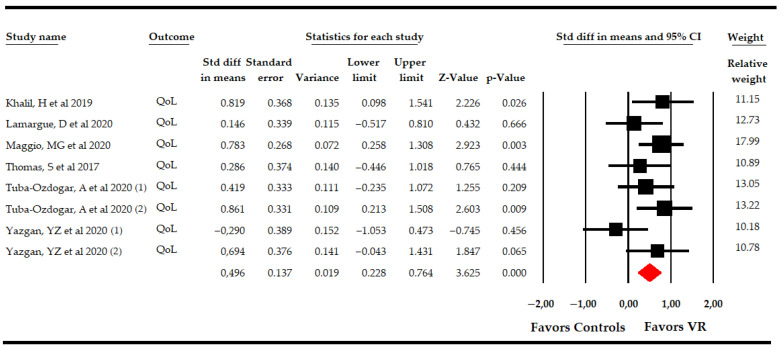
Forest Plot of the Effect of Virtual Reality on Overall Quality of Life.

Six RCTs [49,50,51,53,57,59] reported data from 291 PwMS to assess the effect of VR-based intervention on overall QoL in comparison to CT or simple observation. An initial overall analysis provided moderate-quality evidence of a medium effect favoring VR-based therapy (SMD 0.5; 95% CI 0.23, 0.76; *p* < 0.001) (Table 4, Figure 6), without heterogeneity (I^2^ 2%; Q-test = 7.1, df = 5; *p* 0.21) and no risk of publication bias (*p* for Egger test 0.2) (Supplementary Figure S3). The one study removed showed a variation of 20% with respect to the original pooled effect when a study by Yazgan [53] was excluded.

**Figure 7:**
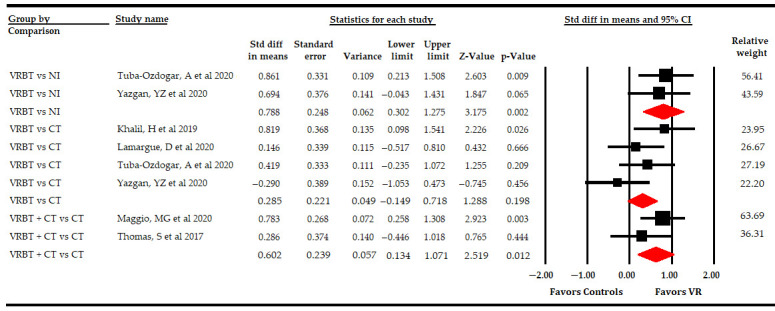
Subgroup Analysis of the Effect of Virtual Reality on Overall Quality of Life According to Specific Comparisons.

On the overall QoL, we checked the effect of VR-based therapy according to the specific comparison (Table 4, Figure 7). Firstly, two studies [51,53] reported data comparing the effect of VR-based intervention vs. simple observation that showed low-quality evidence of a large effect favoring VR-based intervention (SMD 0.79; 95% CI 0.3, 1.28; *p* 0.002), without heterogeneity (I^2^ 0%; Q-test = 0.1, df = 3; *p* 0.75). Secondly, two studies [57,59] compared VR-based intervention with CT vs. CT, showing low-quality evidence of a medium effect (SMD 0.6; 95% CI 0.13, 1.07; *p* 0.012) favoring VR-based intervention with CT without heterogeneity (I^2^ 2%; Q-test = 1, df = 1; *p* 0.32). However, no statistically significant differences were found between VR-based intervention and CT (SMD 0.29; 95% CI −0.15, 0.72; *p* 0.2), without heterogeneity (I^2^ 3.5%; Q-test = 3.1, df = 3; *p* 0.21), in data reported by four studies [49,50,51,53].

##### 3.5.4 Effect of Virtual Reality-Based Therapy on the Physical Dimension of Quality of Life

**Figure 8:**
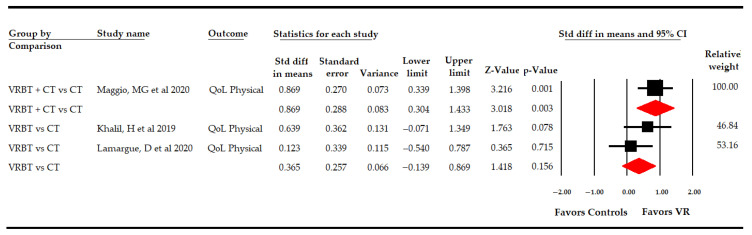
Subgroup Analysis of the Effect of Virtual Reality on the Physical Dimension of Quality of Life, According Specific Comparisons.

Three studies [49,50,59] provided data to assess the effect of VR-based intervention on the physical dimension of QoL (Table 4, Figure 8). In a preliminary analysis, low-quality evidence of a medium effect (SMD 0.58; 95% CI 0.13, 1.02; *p* 0.011) was found favoring VR-based intervention, without heterogeneity (I^2^ 0%; Q-test = 1.9, df = 2; *p* 0.38) and without risk of publication bias (*p* for Egger test 0.55). Specifically, in subgroup analysis, one study [59] compared VR + CT vs. CT, showing low-quality evidence of a large effect (SMD 0.87; 95% CI 0.3, 1.43; *p* 0.003) favoring VR + CT. Finally, when VR-based intervention was compared with CT in two studies [49,50], no statistically significant differences were found between groups (SMD 0.37; 95% CI −0.14, 0.87; *p* 0.16) without heterogeneity (I^2^ 0%; Q-test = 1, df = 1; *p* 0.32).

##### 3.5.5 Effect of Virtual Reality-Based Therapy on the Mental Dimension of Quality of Life

**Figure 9:**
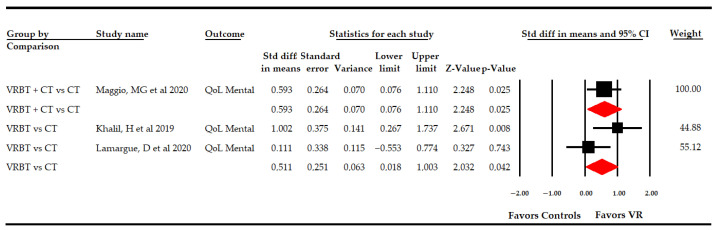
Subgroup Analysis of the Effect of Virtual Reality on the Mental Dimension of Quality of Life According to Specific Comparisons.

Three studies [49,50,59] reported data to assess the effect of VR-based intervention on the mental dimension of QoL (Table 4, Figure 9). Low-quality evidence of a medium effect favoring VR-based intervention (SMD 0.55; 95% CI 0.09, 1.01; *p* 0.018), without low heterogeneity (I^2^ 6.2%; Q-test = 2.1, df = 2; 0.35) and with a possible risk of publication bias, (*p* for Egger test 0.45 and 31% of variation after trim-and-fill estimation) was shown. In subgroup analysis, one study [59] showed low-quality evidence of a medium effect favoring VR + CT (SMD 0.6; 95% CI 0.08, 1.11; *p* 0.025) when compared with CT. Moreover, when VR-based intervention was compared with CT in two studies [49,50], low-quality evidence of a medium effect (SMD 0.51; 95% CI 0.02, 1; *p* 0.042) was shown favoring VR-based intervention, without heterogeneity (I^2^ 0%; Q-test = 1, df = 1; *p* 0.32).

## 4. Discussion

In recent years, some studies have assessed the efficacy of different therapies to reduce the impact of MS and its muscle fatigue symptoms, as well as to increase the QoL of MS patients [60,61]. VR-based intervention is a novel therapy that is being used more in the treatment of neurological diseases [18,62], including MS. To date, a number of published reviews have assessed the effect of VR-based intervention in PwMS regarding balance, gait [29,30], and upper extremity recovery [31]. In addition, a recent review [63], which included a small number of studies, evaluated the effect of VR-based intervention on fatigue and QoL. For this reason, the present work was conceived to compile and analyze the more updated and recent evidence available thus far on the efficacy of VR-based therapy regarding such variables. Our systematic review with meta-analysis includes 12 RCTs published in the last 9 years that provide data from 606 patients with MS (42.83 ± 6.86 years), and evaluate the effect of VR-based intervention on fatigue (7 studies), the impact of MS (5 studies), and QoL (6 studies), differentiating between physical and mental dimensions. In the studies included we identified three different comparisons: (1) VR vs. NI; (2) VR vs. CT; and (3) VR + CT vs. CT. In an overall analysis, our findings showed that VR-based intervention reduces the level of fatigue and the impact of MS, and increases the QoL in PwMS. Specifically, the subgroup analysis revealed that: (1) compared with simple observation, VR-based intervention may be effective to minimize the impact of MS and to increase the overall QoL; (2) VR-based intervention reduces fatigue more than CT; and (3) VR-based intervention with CT is more effective that isolated CT to increase the physical and mental dimensions of the QoL.

Regarding fatigue, approximately 15% of PwMS consider fatigue as the most frequent and disabling symptom that reduces QoL and personal autonomy [64]. Therefore, it is important to find therapies that are able to reduce it. In this case, our overall analysis showed that VR-based intervention produces a low-to-medium effect in reducing fatigue in PwMS. In addition, when subgroup analysis was conducted, we found that VR was better than CT at reducing this variable. Our findings are in line with the recent review of Santos-Nascimento [63], although our meta-analysis on the effect of VR-based interventions on fatigue, includes seven studies (five more than the previous review), which means that our review is able to estimate the effect with data from different tests using Cohen’s SMD, increasing the generalization and quality of evidence of the effect of VR vs. CT in reducing fatigue. However, no statistically significant differences were found when VR was compared with simple observation and when VR-based intervention was used with CT compared to CT alone. It is important to note that these two subgroups included a small number of studies (three and one, respectively) and it is possible that these results will change if new studies are included. It is accepted that in order to develop new therapies that improve muscle strength, muscle oxygenation, and heart function parameters, it is also important to reduce fatigue. Physical exercise has been postulated as an excellent therapy to reduce fatigue as consequence of the improvements in muscle resistance, heart rate, and respiratory frequency, all of which increase the physical condition of patients [65,66]. However, the level of adherence is sometimes low, and PwMS face some barriers such as low functional capability, fear of falling, or difficulties in attending rehabilitation centers. VR-based interventions are based on the active performance of physical and functional exercises through video games that can be adapted in intensity, which means that the characteristics of a VR-based exercise can be adapted to take into account the patient’s state of fatigue. This enables continuous training, even at home, increasing patient motivation and thus, probably, the effectiveness of VR therapy [67]. In addition, increasing patient motivation in VR therapy may increase their adherence to the therapy, thus favoring continuous training to improve muscular endurance and reduce movement fatigue [67]. This high level of adherence to VR therapy may be one of the causes of major improvements in fatigue in a VR group compared to a CT group. Sometimes, classical neurorehabilitation protocols are based on monotonous and passive CT exercises, while VR-based intervention allows for an enjoyable and customized therapy that involves the active immersion of patients and continuous work.

Our meta-analysis demonstrates that the use of VR-based therapies in neurorehabilitation protocols reduces the disabling impact of MS symptoms, although with a low effect. Subgroup analysis reported a medium effect of VR-based intervention compared to simple observation. However, no statistically significant differences were observed when VR-based intervention was compared to CT, as well as when VR-based intervention was used in combination with CT vs. CT alone. These results should be generalized with caution, due to the small number of studies included in each analysis. However, our results suggest that, in the absence of physical therapy, VR-based intervention alone can be used as an effective therapy to reduce the disabling impact of MS. In addition, it is important to remark that this study is the first review with a meta-analysis that provides information about the effect of VR-based interventions on the impact of MS.

Finally, we assessed the effect of VR-based interventions on global QoL. In a preliminary meta-analysis, VR-based intervention improves global QoL with a medium effect in PwMS. Subgroup analysis showed that VR and VR + CT are better than simple observation and CT, respectively, at improving overall QoL in PwMS. Compared to simple observation, our results are in line with the meta-analysis conducted by Santos-Nascimiento [63], although we include four more studies in our analysis. Both reviews are in favor of using VR-based therapy in CT protocols to improve the overall QoL. However, no statistically significant differences were found when VR-based intervention was compared with CT. We suggest that both therapies (VR or CT), when are used as single therapeutic options, are effective; but when these two therapies are combined, the effect is significantly greater than CT alone. Furthermore, we performed a subgroup analysis to assess the effect of VR-based intervention on the physical and mental dimensions of QoL, which was the first meta-analysis to have explored this factor. In both dimensions, the greatest effect of VR-based therapy was found when it was used in combination with CT compared to CT alone, with the important limitation that this analysis included one study. Although this result is limited, it is one of the most important findings of this review, as it reinforces the idea that VR-based intervention combined with CT can increase the effect of both therapies on QoL. In this sense, QoL can be improved thanks to the combination of these two therapies with two different objectives. As such, CT is a therapy more focused on analytical movements, while VR-based therapy allows PwMS to carry out functional movements and train activities for daily living with active exercise-based videogames integrated in sessions that are more ludic and motivating. Combining the use of customized CT techniques to restore specific joint, muscle, or balance disorders, together with VR-based intervention of different levels of difficulty and adapted to patient’s preferences, can explain this large improvement in QoL metrics. Several studies have reported higher levels of fun and commitment in therapy in patients receiving a VR-based intervention compared to CT alone [68]. These positive results, along with improvements in mental overload during VR exposure, could be responsible for the increase in outcomes such as QoL [68]. Furthermore, in older adults, VR-based intervention can produce changes in the hippocampus and amygdala, which could be related to the control of negative emotions and, therefore, help reduce anxiety and depression; this approach could be applied to improve the QoL in PwMS [69]. The results of our review are in line with studies carried out for different neurological diseases [70,71,72], including MS [54], which show an improvement in QoL metrics after VR-based therapy. Finally, our systematic review includes a large number of studies that assess the effect of VR-based intervention on QoL, which increases the quality of evidence in comparison to other reviews [63].

At a neurophysiological level, VR-based interventions look to promote neuronal plasticity in the damaged brains of PwMS, with the aim of replacing or restoring missing functions. The brain is capable of adapting to environmental and pathological stimuli through neuronal or cerebral plasticity [73]. In MS, remyelination is essential to repair demyelination and recover from disabling symptoms [74], and neuronal plasticity is necessary to reorganize new synapses, with remyelination being responsible for clinical improvement in MS [75]. Neuronal plasticity decreases with age and with the duration of MS [76], so it is important to apply active and multisensory therapies in the first years of MS diagnosis, when patients are young. The multisensory experience produced by VR and the active participation of the patients to perform the therapy through videogames [23] could activate the mirror neuron system (MNS). The MNS, located in the frontal (inferior frontal gyrus) and parietal (inferior parietal lobe) lobes [77], is activated during the execution of a motor action and when an individual observes an action in other subjects [78]. Functional movements carried out with VR devices or the visualization of movements in VR devices could facilitate the activation of MNS in PwMS, possibly producing cortical and subcortical brain changes that stimulate synaptic remyelination and reorganization in motor brain areas. Furthermore, some studies have suggested that VR increases the motivation of MS patients during therapy, as well as their adherence to therapy [79]. In addition to active participation and enjoyment, the effect produced by VR-based intervention may be related to a distraction strategy. Previous studies have shown the distracting power of VR for the treatment of pain and anxiety in different situations due to immersion in virtual environments [80]. Compared other classical therapies, the distraction produced by a VR-based intervention focuses a patient’s attention in the videogames, reduces negative emotions such as anxiety [81], and increases participation in the therapy. The power of distraction could be related to the effect of VR on the prefrontal cortex, which is responsible for blocking negative experiences and feelings [82]. The prefrontal cortex, specifically the dorsolateral prefrontal cortex and the inferior frontal gyrus, plays a crucial role in the inhibition of emotive responses and may be related to the regulation of emotions [83]. Thus, VR could be considered an excellent option to improve the mental dimension of QoL in PwMS who have difficulties adhering to CT.

This review presents updated practical implications for physical clinicians, such as physiotherapists or occupational therapists, as well as researchers. It also shows how VR-based therapy can reduce disabling symptoms of MS such as fatigue and increase QoL. The main advantage of VR-based intervention is the possibility of obtaining virtual environments that PwMS feel are similar to the real world, which leads them to perform functional tasks within these environments. VR-based intervention also has the added value of allowing participants to interact dynamically with objects or situations that would not be possible in the real world, promoting motor learning [84] with augmented feedback and multisensory inputs. Furthermore, VR-based intervention is a safe technique with few adverse effects reported in subjects with MS [85], and it offers the possibility of home treatment, a relevant advantage during the COVID-19 pandemic [86]. Various systematic reviews have demonstrated the efficacy of VR-based intervention as a home training method in the COVID-19 pandemic in patients with different neurological diseases, including MS [86,87]. Scientific evidence shows that home training based on VR is a therapy that provides motivation, and it can be useful in the rehabilitation of physical and cognitive function in PwMS [88]. The use of VR at home seems to have a positive impact as a method of support for traditional rehabilitation, especially during the COVID-19 pandemic, due to the difficulty of these patients accessing classical therapies in clinical centers [89]. For example, physical exercise improves resistance to fatigue and QoL, and can be practiced at home through VR-based videogames exercises. In this sense, the most analyzed systems used in VR neurorehabilitation are niVR devices, such as Nintendo^®^ Wii Balance Board^®^ or Nintendo^®^ Wii Fit^®^, which are affordable and easier to transport and install at home. Other VR systems, such as BTS-Nirvana or Oculus Quest, allow full 360° immersion in the virtual world, but also require a high level of spatial orientation and comprehension [90], and are more appropriate for use in clinical centers supervised by a clinician. In our review, the majority of studies (10 of 12) included niVR devices; thus, these results are more dependent on non-immersive virtual scenarios, which may be the most useful for clinical practice and home training in PwMS.

Finally, we must bear in mind that the present work has some limitations, and the results should be interpreted with caution. First, the low number of studies included in each meta-analysis and in the subgroup analyses, as well as the low number of participants per study, reduces the generalizability of our findings. Second, the low methodological quality of the included studies increases the risk of selection and classification bias. Third, the presence of publication bias and the variation in trim-and-fill estimations may distort the real effect of the therapy for different outcomes. Another limitation comes from the large variations observed in the sensitivity analysis, which may reduce the quality of our findings. In addition, the majority of the studies assessed the effect of VR-based intervention using niVR devices, so our results are more relevant to the effect of niVR devices. Finally, we must remark that all the assessments conducted in the included studies were performed immediately after intervention, which did not permit us to predict the effect of VR-based therapy in the medium and long term.

## 5. Conclusions

Our results showed that VR-based therapy is effective in reducing fatigue and the impact of MS, as well as increasing QoL in PwMS. Specifically, to reduce fatigue, VR-based intervention is better than CT. In terms of the impact of MS, VR-based intervention was better than simple observation. To increase overall QoL, VR-based therapy is better than simple observation and the combined use of VR-based intervention with CT is better than CT alone. Finally, VR-based intervention also showed a positive effect on the physical and mental dimensions of QoL, demonstrating a significant increase in both dimensions when the VR-based intervention was used in combination with CT, compared to CT alone. Nevertheless, further research is needed to assess the effect of VR-based intervention, both alone and when combined with other therapies.

## Figures and Tables

**Table 1:** Search strategy in each database.

Database	Search Strategy
PubMed Medline	(multiple sclerosis[mh] or multiple sclerosis[tiab] or “multiple sclerosis”[tiab]) AND (virtual reality[mh] OR virtual reality[tiab] OR virtual reality exposure therapy[mh] OR virtual reality exposure therapy[tiab] OR “virtual reality”[tiab] OR videogames[tiab] OR exergames[tiab] OR serious games[tiab]) AND (fatigue[mh] OR “fatigue”[tiab] OR muscle fatigue[mh] OR muscle fatigue[tiab] OR quality of life[mh] OR quality of life[tiab])
SCOPUS	(TITLE-ABS-KEY (“multiple sclerosis” OR “esclerosis múltiple”) AND TITLE-ABS-KEY (“virtual reality” OR “videogames” OR “exergames” OR “serious games” OR “games”) AND TITLE-ABS-KEY (“fatigue” OR “quality of life”))
Web of Science	(*multiple sclerosis* OR *esclerosis múltiple*) (Topic) and (*virtual reality* OR *exergames* OR * videogames* OR *serious games* OR *games*) (Topic) and (*fatigue* OR *quality of life*) (Topic)
PEDro	(multiple sclerosis) and (virtual reality)

**Table 2:** Characteristics of the Studies Included in the Review.

					VR Group				Control Group				Outcomes	
Study	Design	Country	Funding	K	N	Age	F:M	Intervention	N	Age	F:M	Intervention	Outcomes	Test
Brichetto, G. et al., 2013 [48]	Pilot RCT	Italy	No	1	18	40.7	10:8	12 sessions of Nintendo^®^ Wii Balance Board^®^ during 4 weeks, 3 sessions per week. Each sessions lasted 60 min	18	43.2	12:6	CT (Static and dynamic exercises in single and double leg stance)	Fatigue	M-FIS
Cuesta-Gómez, A. et al., 2020 [54]	RCT	Spain	Yes	2	16	49.8	9:7	20 sessions using serious games with LMC, during 10 weeks, 2 sessions per week of 60 min. Conventional Therapy, for 60 min, in upper extremity was added	14	42.6	9:5	CT (Physiotherapy motor rehabilitation of upper extremity joints using mobilizations, stretching and functional tasks)	Fatigue	FSS
													Impact	MSIS-29
Khalil, H. et al., 2019 [49]	Pilot RCT	Jordan	Yes	2	16	39.8	12:4	Exercises in different niVR scenarios (Nintendo^®^ Wii Balance Board^®^), during 6 weeks, 2 sessions per week	16	34.8	10:6	CT (Balance exercises)	Fatigue	M-FIS
													QoL	SF-36
Lamargue, D et al., 2020 [50]	RCT	France	Yes	2	18	43.8	12:6	REACTIV program based niVR, during 4 months, 3 sessions per week. Each session lasted 45 min.	17	38.3	14:3	CT (Physical activity and global cognitive stimulation)	Fatigue	M-FIS
													QoL	SF-36
Maggio, M.G. et al., 2020 [59]	RCT	Italy	NR	1	30	51.9	12:18	Semi-immersive VR using BTS-Nirvana for a total of 24 sessions using niVR, during 8 weeks, 3 sessions per week. Each session lasted 60 min. In addition, CT program was added	30	48.2	17:13	CT (General conditioning exercises of strengthening, gait and postural control)	QoL	MS-QoL
Ozkul, C. et al., 2020 [52]	RCT	Turkey	No	2	13	29	9:4	Exercises using augmented and iVR (RAGU system) during 8 weeks, 2 sessions per week. A total of 16 sessions, 20 min each.	13	34	11:2	CT (balance training trough exercises with ball)	Fatigue	FSS
									13	34	10:3	NI (Simple observation)		
Prosperini, L. et al., 2013 [55]	RCT	Italy	No	2	18	35.3	13:5	Home-based training with Nintendo^®^ Wii Balance Board^®^ during 12 weeks, 5 sessions per week. Sessions lasted 30 min.	18	37.1	12:6	NI (Simple observation)	Impact	MSIS-29
					18	37.1	12.6		18	35.3	13:5	NI (Simple observation)		
Robinson, J. et al., 2015 [56]	RCT	UK	Yes	2	20	52.6	14:6	8 sessions of exercises using Nintendo^®^ Wii Fit videogames during 4 weeks, 2 sessions per week. Sessions lasted 40–60 min	16	53.9	12:4	CT (Balance training)	Impact	WHODAS 2.0
									15	51.9	12:3	NI (Simple observation)		
Thomas, S. et al., 2017 [57]	RCT	UK	Yes	2	15	50.9	14:1	Home-based training and personalized Nintendo^®^ Wii Balance Board^®^ using Mii-vitaleSe, in addition to other therapies, including CT, as medical treatment if patients require it.	15	47.6	13:2	CT (usual care, physical, medicine and education support)	Impact	MSIS-29
													QoL	SF-36
Tollár, J. et al., 2019 [58]	RCT	Hungary	No	2	14	48.2	12:2	25 sessions using Xbox 360 and Kinect sensor, during 5 weeks, 5 sessions per week. Each session lasted 1 h.	14	46.9	12:2	CT (Dynamic and static balance exercises	Impact	MSIS-29
									12	47	11:1	NI (Simple observation)		
Tuba-Ozdogar, A. et al., 2020 [51]	RCT	Turkey	Yes	4	20	39.2	16:4	8 sessions using Microsoft Xbox One and Kinect motion sensor, during 8 weeks, 1 session per week (45 min per session)	17	43.6	12:5	CT (Balance, stretching and core stability exercises)	Fatigue	M-FIS
									20	37.9	15:5	NI (Simple observation)	QoL	MS-QoL
Yazgan, Y.Z. et al., 2020 [53]	RCT	Turkey	Yes	4	15	47.4	13:2	16 sessions of exercises using Nintendo^®^ WiFit videogames during 8 weeks, 2 sessions per week. Sessions lasted 60 min	12	43.1	12	CT (Balance training exercises)	Fatigue	FSS
									15	40.6	13:2	NI (Simple observation)	QoL	MS-QoL

Abbreviations: K = number of independent comparisons; RCT = randomized controlled trial; N = number of participants; F = female; M= male; niVR = non-immersive virtual reality; LMC = Leap Motion Controller; iVR = immersive virtual reality; CT = conventional therapy; NI = no intervention (simple observation); UK = United Kingdom; QoL = quality of life; FSS = Fatigue Severity Scale; MFIS = Modified Fatigue Impact Scale; MSIS-29 = Multiple Sclerosis Impact Scale-29; MS-QoL = Multiple Sclerosis Quality of Life; WHODAS = World Health Organization Disability Assessment Schedule 2.0.

**Table 3:** Score of Included Studies from PEDro Assessment.

Study	i1	i2	i3	i4	i5	i6	i7	i8	i9	i10	i11	Total
Brichetto, G. et al., 2013 **[48]**	Yes	Yes	No	Yes	No	No	Yes	No	No	Yes	Yes	5/10
Cuesta-Gómez, A. et al., 2020 [54]	Yes	Yes	No	Yes	No	No	Yes	Yes	No	Yes	Yes	6/10
Khalil, H. et al., 2019 **[49]**	Yes	Yes	Yes	Yes	No	No	Yes	No	No	Yes	Yes	6/10
Lamargue, D. et al., 2020 [50]	Yes	Yes	No	Yes	No	No	Yes	Yes	No	Yes	Yes	6/10
Maggio, M.G. et al., 2020 [59]	Yes	Yes	Yes	Yes	No	No	Yes	Yes	No	Yes	Yes	7/10
Ozkul, C. et al., 2020 [52]	Yes	Yes	No	Yes	No	No	No	Yes	No	Yes	Yes	5/10
Prosperini, L. et al., 2013 [55]	Yes	Yes	Yes	Yes	No	No	No	Yes	No	Yes	Yes	6/10
Robinson, J. et al., 2015 [56]	Yes	Yes	No	Yes	No	No	No	No	Yes	Yes	Yes	5/10
Thomas, S. et al., 2017 [57]	Yes	Yes	Yes	Yes	No	No	No	Yes	Yes	Yes	Yes	7/10
Tollár, J. et al., 2019 [58]	Yes	Yes	Yes	Yes	No	No	Yes	Yes	No	Yes	Yes	7/10
Tuba-Ozdogar, A. et al., 2020 [51]	No	Yes	No	Yes	No	No	No	Yes	No	Yes	Yes	5/10
Yazgan, Y.Z. et al., 2020 [53]	Yes	Yes	No	Yes	No	No	No	Yes	No	Yes	Yes	5/10

Abbreviations: i1 = eligibility criteria; i2 = random allocation; i3 = concealed allocation; i4 = baseline comparability; i5 = blind subjects; i6 = blind therapists; i7 = blind assessors; i8 = adequate follow-up; i9 = intention-to-treat analysis; i10 = between-group comparisons; i11 = point estimates and variability.

**Table 4:** Main Findings in Meta-Analyses.

						Effect Size			Publication Bias			Heterogeneity		
Outcomes	Groups		K	N	N_s_	SMD	95% CI	*p*	Funnel Plot	Trim-and-Fill		Q-test	I^2^	*p*
										Adjusted SMD	% of Var.			
Fatigue	Overall Analysis		10	319	31.9	−0.33	[−0.61, −0.06]	0.02	Symmetric	−0.33	0%	9	0%	0.44
	Specific comparison subgroups	VR vs NI	3	96	33	−0.2	[−0.61, 0.2]	0.33	Symmetric	−0.22	0%	1.9	0%	0.37
		VR vs CT	6	193	32.2	−0.4	[−0.7, −0.11]	0.006	Asymmetric	−0.51	27%	5.1	1.9%	0.41
		VR + CT vs CT	1	30	30	−0.23	[−0.95, 0.48]	0.52	NP	NP	NP	NP	NP	NP
Impact of Multiple Sclerosis	Overall Analysis		8	287	31.8	−0.3	[−0.55, −0.04]	0.02	Asymmetric	−0.61	50%	8.9	21%	0.26
	Specific comparison subgroups	VR vs NI	4	103	25.8	−0.61	[−0.97, −0.23]	0.001	Asymmetric	−0.74	23%	5.5	27.7%	0.23
		VR vs CT	2	64	32	−0.03	[−0.48, 0.53]	0.92	NP	NP	NP	0.98	0%	0.32
		VR + CT vs CT	2	90	45	−0.02	[−0.54, 0.49]	0.93	NP	NP	NP	0.3	0%	0.6
Overall Quality of Life	Overall Analysis		8	291	36.3	0.5	[0.23, 0.76]	<0.001	Symmetric	0.5	0%	7.1	2%	0.21
	Specific comparison subgroups	VR vs NI	2	99	49.5	0.79	[0.3, 1.28]	0.002	NP	NP	NP	0.1	0%	0.75
		VR vs CT	4	166	41.5	0.29	[−0.15, 0.72]	0.2	Asymmetric	0.44	57%	3.1	3.5%	0.21
		VR + CT vs CT	2	90	45	0.6	[0.13, 1.07]	0.012	NP	NP	NP	1	0%	0.32
Physical Quality of Life	Overall Analysis		3	127	42.3	0.58	[0.13, 1.02]	0.011	Symmetric	0.58	0%	1.9	0%	0.38
	Specific comparison subgroups	VR vs CT	2	67	33.5	0.37	[−0.14, 0.87]	0.16	NP	NP	NP	1	0%	0.32
		VR + CT vs CT	1	60	60	0.87	[0.3, 1.43]	0.003	NP	NP	NP	NP	NP	NP
Mental Quality of Life	Overall Analysis		3	127	42.3	0.55	[0.09, 1.01]	0.018	Asymmetric	0.38	31%	2.1	6.2%	0.35
	Specific comparison subgroups	VR vs CT	67	33.5	33.5	0.51	[0.02, 1]	0.042	NP	NP	NP	1	0%	0.32
		VR + CT vs CT	60	60	60	0.6	[0.08, 1.11]	0.025	NP	NP	NP	NP	NP	NP

Abbreviations: K = number of independent comparisons; N = number of participants; N_s_ = mean number of participants per study; SMD = Cohen’s Standardized Mean Difference; 95% CI = 95% confidence interval; *p* = *p*-value; % of var = percentage of variation; I^2^ = degree of inconsistency; VR = virtual reality; NI = not intervention; CT = conventional therapy; NP = not possible.

## Supplementary Materials

The following are available online at https://www.mdpi.com/article/10.3390/s21217389/s1, Figure S1. Funnel Plot of the Effect of Virtual Reality on Fatigue. Figure S2. Funnel Plot of the Effect of Virtual Reality on Multiple Sclerosis Impact. Figure S3. Funnel Plot of the Effect of Virtual Reality on Overall Quality of Life. Table S1. Independent Comparisons Provided by Each Study Included in the Review.
