# Supplementary material for: Virtual Reality-Based Therapy Improves Fatigue, Impact, and Quality of Life in Patients with Multiple Sclerosis. A Systematic Review with a Meta-Analysis
Source: Sensors (Basel). 2021 Nov 6;21(21):7389. doi: 10.3390/s21217389 (PMC8588272; doi:10.3390/s21217389)
Supplement: Supplementary file 1 [file sensors-21-07389-s001.zip › sensors-1399553-supplementary/Suplementary Figures.pdf]

SUPPLEMENTARY FIGURES

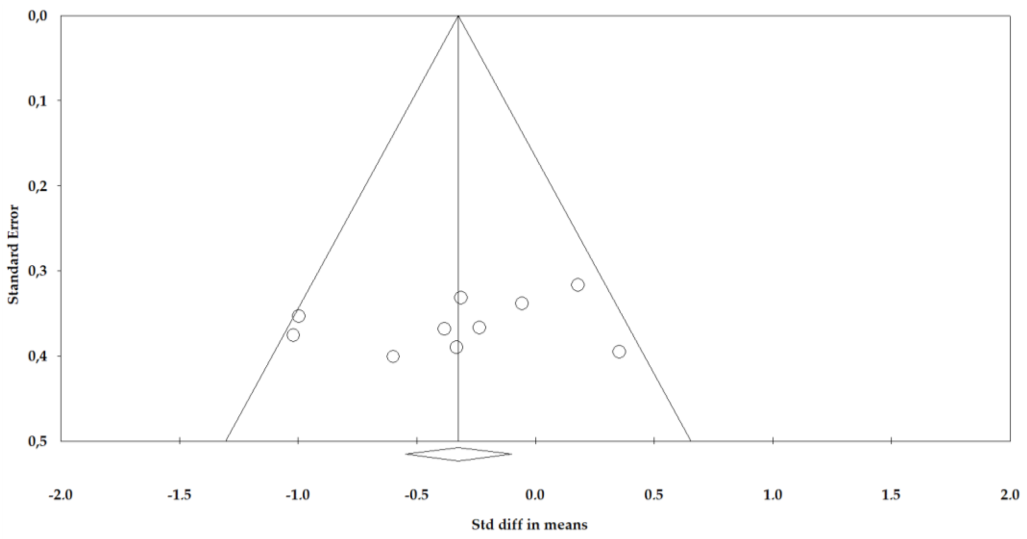

Figure S1. Funnel Plot of the Effect of Virtual Reality on Fatigue. Note: White points = Initial studies included; White Diamond = Effect size without Trim-and-fill estimation.

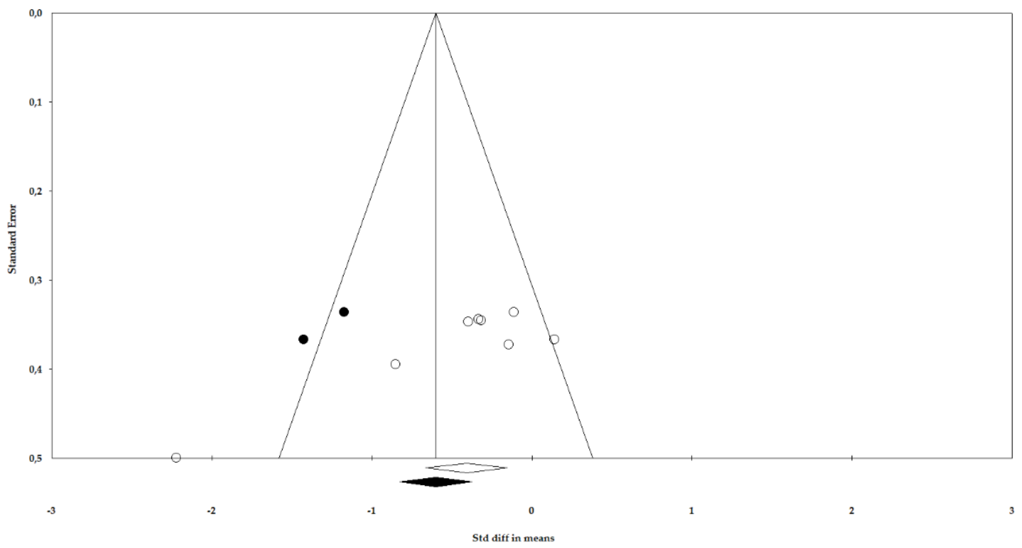

Figure S2. Funnel Plot of the Effect of Virtual Reality on Multiple Sclerosis Impact. Note: Black points = Imputed studies with Trim-and-fill estimation; Black diamond = Adjusted effect size with Trim-and-fill estimation; White points = Initial studies included; White Diamond = Effect size without Trim-and-fill estimation.

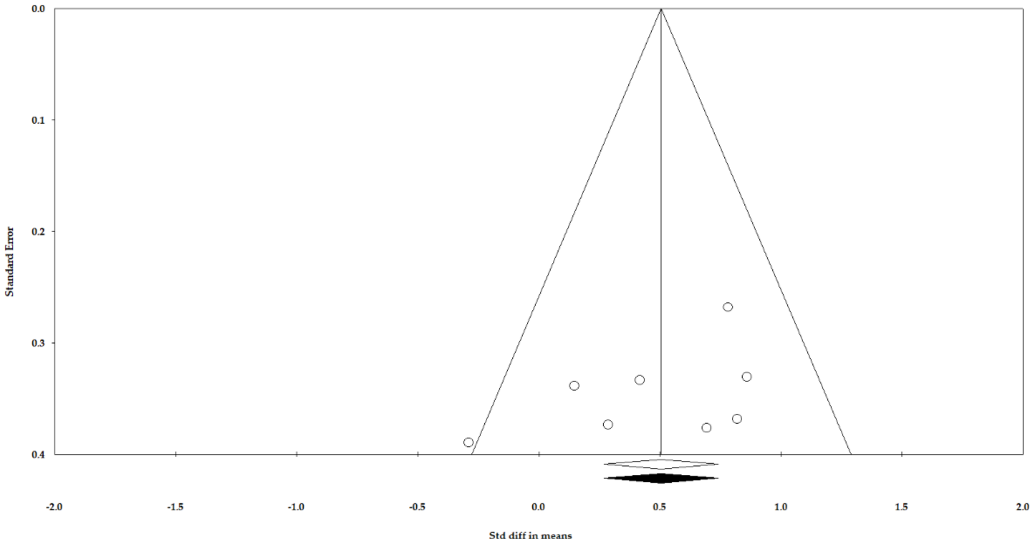

Figure S3. Funnel Plot of the Effect of Virtual Reality on Global Quality of Life. Note: Black diamond = Adjusted effect size with Trim-and-fill estimation; White points = Initial studies included; White Diamond = Effect size without Trim-and-fill estimation.
