# Supplementary material for: Virtual Reality-Based Therapy Improves Fatigue, Impact, and Quality of Life in Patients with Multiple Sclerosis. A Systematic Review with a Meta-Analysis
Source: Sensors (Basel). 2021 Nov 6;21(21):7389. doi: 10.3390/s21217389 (PMC8588272; doi:10.3390/s21217389)
Supplement: Supplementary file 1 [file sensors-21-07389-s001.zip › sensors-1399553-supplementary/Table S1. Independent Comparisons.pdf]

Table S1. Independent Comparisons Provided by Each Study Included in the Review

Study	Number of Groups	Number of Comparisons	Independent Comparisons	Outcome
Brichetto, G et al 2013 [48]	2	1	VRBT vs CT	Fatigue
Cuesta-Gómez, A et al 2020 [56]	2	2	VRBT vs CT	Fatigue
			VRBT vs CT	Impact
Khalil, H et al 2019 [49]	2	2	VRBT vs CT	Fatigue
			VRBT vs CT	Impact
Lamargue, D et al 2020 [52]	2	2	VRBT vs CT	Fatigue
			VRBT vs CT	Quality of Life
Maggio, MG et al 2020 [51]	2	1	VRBT vs CT	Quality of Life
Ozkul, C et al 2020 [54]	3	2	VRBT vs CT	Fatigue
			VRBT vs NI	Fatigue
Prosperini, L et al 2013 [57]	2	2	VRBT vs NI	Impact
			VRBT vs NI	Impact
Robinson, J et al 2015 [58]	3	2	VRBT vs CT	Impact
			VRBT vs NI	Impact
Thomas, S et al 2017 [59]	2	2	VRBT vs CT	Impact
			VRBT vs CT	Quality of Life
Tollár, J et al 2019 [50]	3	2	VRBT vs CT	Impact
			VRBT vs NI	Impact
Tuba-Ozdogar, A et al 2020 [53]	3	4	VRBT vs CT	Fatigue
			VRBT vs NI	Fatigue
			VRBT vs CT	Quality of Life
			VRBT vs NI	Quality of Life
Yazgan, YZ et al 2020 [55]	3	4	VRBT vs CT	Fatigue
			VRBT vs NI	Fatigue
			VRBT vs CT	Quality of Life
			VRBT vs NI	Quality of Life

Abbreviations: VRBT = Virtual Reality-based Therapy; CT = Conventional Therapy; NI = No intervention

Note: *In Prosperini study we identified two groups (VR and NI) obtaining the first comparison about impact of MS. Later, both groups are crossed returning to have a new comparison about impact of MS.
